# Comparative Transcriptomics Among Four White Pine Species

**DOI:** 10.1534/g3.118.200257

**Published:** 2018-03-27

**Authors:** Ethan A. G. Baker, Jill L. Wegrzyn, Uzay U. Sezen, Taylor Falk, Patricia E. Maloney, Detlev R. Vogler, Annette Delfino-Mix, Camille Jensen, Jeffry Mitton, Jessica Wright, Brian Knaus, Hardeep Rai, Richard Cronn, Daniel Gonzalez-Ibeas, Hans A. Vasquez-Gross, Randi A. Famula, Jun-Jun Liu, Lara M. Kueppers, David B. Neale

**Affiliations:** * Department of Ecology and Evolutionary Biology, University of Connecticut, Storrs, CT; † Department of Plant Pathology, University of California, Davis, CA; ‡ USDA-Forest Service, Pacific Southwest Research Station, Institute of Forest Genetics, Placerville, CA; § Department of Ecology and Evolutionary Biology, University of Colorado, Boulder, CO; ** USDA-Forest Service Pacific Southwest Research Station, Davis, CA; †† US Department of Agriculture, Agricultural Research Service, Horticultural Crop Research Unit, Corvallis, OR; ‡‡ Department of Biology, Utah State University, Logan, UT 84322; §§ USDA-Forest Service Pacific Northwest Research Station, Corvallis, OR; *** Department of Plant Sciences, University of California, Davis, CA; ††† Pacific Forestry Center, Canadian Forest Service, Natural Resources Canada, Victoria, BC, Canada; ‡‡‡ Sierra Nevada Research Institute, University of California, Merced, California 95343; §§§ Earth Sciences Division, Lawrence Berkeley National Laboratory, Berkeley, CA 94720

**Keywords:** *de novo* transcriptome assembly, white pines, five-needle pines, wpbr, *Pinus monticola*, *Pinus flexilis*, *Pinus albicaulis*, *Pinus lambertiana*

## Abstract

Conifers are the dominant plant species throughout the high latitude boreal forests as well as some lower latitude temperate forests of North America, Europe, and Asia. As such, they play an integral economic and ecological role across much of the world. This study focused on the characterization of needle transcriptomes from four ecologically important and understudied North American white pines within the *Pinus* subgenus *Strobus*. The populations of many *Strobus* species are challenged by native and introduced pathogens, native insects, and abiotic factors. RNA from the needles of western white pine (*Pinus monticola*), limber pine (*Pinus flexilis*), whitebark pine (*Pinus albicaulis)*, and sugar pine (*Pinus lambertiana*) was sampled, Illumina short read sequenced, and *de novo* assembled. The assembled transcripts and their subsequent structural and functional annotations were processed through custom pipelines to contend with the challenges of non-model organism transcriptome validation. Orthologous gene family analysis of over 58,000 translated transcripts, implemented through Tribe-MCL, estimated the shared and unique gene space among the four species. This revealed 2025 conserved gene families, of which 408 were aligned to estimate levels of divergence and reveal patterns of selection. Specific candidate genes previously associated with drought tolerance and white pine blister rust resistance in conifers were investigated.

Extant conifers, of the order *Pinales*, represent the largest subset of gymnosperms with seven families, 70 genera, and over 600 species (Christenhusz *et al.* 2011). They are found across North America, Europe, and Asia and are especially dominant in the mountainous mid-continental forests and the boreal forests found at high latitudes on these continents. The species presented in this study, western white pine (*Pinus monticola)*, sugar pine *(Pinus lambertiana)*, limber pine *(Pinus flexilis)* and whitebark pine *(Pinus albicaulis)*, are all five-needle white pines classified as members of the *Pinus* subgenus *Strobus*. Two of these (limber pine and whitebark pine) are considered high elevation species while sugar pine and western white pine range from just above sea level to higher elevations. All four long lived species are found in the mountain ranges of the Western United States and Canada. Sugar pine and limber pine can be found as far south as Northern Baja Mexico (Figure 1).

**Figure 1:**
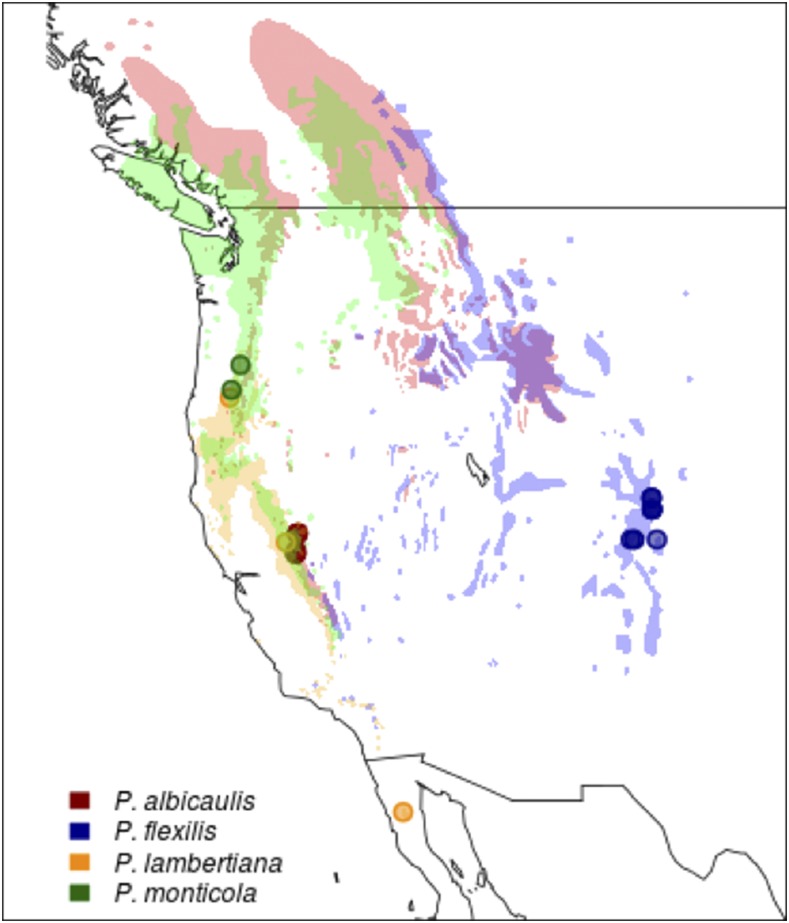
White Pine Range Map and Plant Material Source Locations. Shading indicates typical habitat in western North America for indicated white pine species. Points indicate sampling sites (or common garden sources) for needle tissue used in sequencing.

In North America, white pines are of interest both economically and ecologically. Their role in carbon sequestration, preservation of biodiversity, watershed protection, protracting snowmelt, and soil stabilization is critical (Maloney *et al.* 2012). At present, the white pines are of particular ecological concern due to the prevalence of white pine blister rust (WPBR) caused by the fungus *Cronartium ribicola*, which has had a severe impact on their populations (Liu *et al.* 2013). Introduced to North America in 1900, the fungus, which infects all five-needle white pine species, can result in tree mortality, reduced fecundity, and fitness. Coupled with outbreaks of the native mountain pine beetle (*Dendroctonus ponderosae*), these threats have resulted in severe population declines (Schoettle *et al.* 2013). The northern distributions of whitebark pine and limber pine have been particularly impacted and as such, whitebark pine was listed under the Species at Risk Act as Endangered in Canada in 2012 and by the US Endangered Species Act in 2011.

Among conifers, the white pines have some of the largest genomes. Estimates of genome size range from 27 to 40 Gbp; for reference, the *Arabidopsis* genome is estimated at roughly 157 Mbp (Grotkopp *et al.* 2004, Bennett *et al.* 2003). Current research suggests that the vast difference in the size of *Pinus* genomes is primarily due to amplification of a diverse set of long terminal repeat (LTR) retrotransposons as polyploidy is rarely observed (Morse *et al.* 2009; Kovach *et al.* 2010; Wegrzyn *et al.* 2014). The comparatively massive size of the *Pinus* genomes has historically presented geneticists with a unique set of sequencing and computational challenges. Recent advancements in next generation sequencing (NGS) and assembly approaches has increased the speed and decreased the cost of deep sequencing these large genomes. This has led to the recent publication of the genome of three economically important conifer species: white spruce (*Picea glauca)*, Norway spruce (*Picea abies*), and loblolly pine (*Pinus taeda*) (Birol *et al.* 2013; Nystedt *et al.* 2013; Neale *et al.* 2014). The sugar pine genome, the first white pine, and largest genome sequenced to date, has also been recently characterized (Stevens *et al.* 2016; Crepeau *et al.* 2016). Despite these advances, these complex genomes are comprised of hundreds of thousands to tens of millions of scaffolds and remain available for only a handful of species. As such, many comparative genomics studies focus on the analysis of nuclear genes. The estimated number of unigenes in conifers is surprisingly consistent with the number annotated in their distant and much smaller angiosperm relatives (Soltis and Soltis 2013). Nevertheless, a growing number of RNA-Seq and related comparative studies have asked questions regarding the unique characteristics of the conifer gene space (Buschiazzo *et al.* 2012; Chen *et al.* 2012; De La Torre *et al.* 2015). Transcriptomic profiling is able to generate a tremendous amount of functional information on the coding regions and provide a foundation to apply genomic information to forestry and breeding applications.

To date, limited genetic resources have been made available for the white pines. As a result of extensive efforts in WPBR resistance breeding programs and the availability of the sugar pine genome sequence, some have recently emerged. In this study, we present the first comprehensive analysis of the needle transcriptomes of four white pine species. To provide improved characterization, transcriptomic resources of western white pine were re-assembled from Liu *et al.* (2013). In all four species, RNA from needle tissue of select individuals was sequenced using Illumina short reads and assembled *de novo* into transcriptomes. A comparative study followed to characterize the gene space and identify patterns of selection among orthologous gene families.

## Materials and Methods

### Plant Material, cDNA Library Construction, and Sequencing

Total RNAs were extracted from mature needle tissue from limber pine and whitebark pine using an RNA midi kit (Qiagen, Valencia, CA, USA) and quality was assessed with the Agilent 2100 Bioanalyzer (Agilent Technologies, Santa Clara, CA, USA). The strand-non-specific RNA-seq libraries were constructed from 4 μg of total RNA using the Illumina TruSeq RNA sample preparation Kit (Illumina, San Diego, CA, USA) according to the manufacturer’s protocol, with a library insert size of 300 bp (fragmentation time of 12 min) for paired-end runs. Library profiles were evaluated using an Agilent 2100 Bioanalyzer. RNA extraction and library assembly for sugar pine followed the approach outlined in Hayden *et al.* (2014).

The Illumina GAIIx platform was used for deep 80bp single end sequencing of three cDNA libraries representing mature needle tissue of three independent sugar pine individuals (Center for Genome Research and Biocomputing, Corvallis, OR). The Illumina HiSequation 2000 platform was used to sequence cDNA libraries from both the 5′- and 3′- end of 100-bp reads for both whitebark and limber pine (UC Davis Genome Center, Davis, CA). Whitebark pine samples were pooled into two cDNA libraries with four individuals each representing northern and southern populations around the Lake Tahoe basin. A total of 10 samples were divided into two pooled needle cDNA libraries of five individuals each for the sequencing of limber pine. These two libraries represented high and low elevation populations in the Rocky Mountains. The deconvolution of fluorescent images to DNA sequences, base-calling and quality value calculation were performed using the Illumina data processing pipeline (version 1.4 for Illumina GAIIx and 1.8 for Illumina HiSeq). Read data are publicly available under SRA Accession numbers SRS653581, SRR1506086, SRR1502852, and SRR1506063.

RNA-Seq reads from western white pine were included from Liu *et al.* (2013). The original study examined expression differences in populations of western white pine that were resistant or susceptible to WPBR (Liu *et al.* 2013). For the purpose of the comparative analysis, only the data from the uninfected population (n = 10) was analyzed (SRR1013833, SRR1013836, and SRR1013837). Western white pine needle cDNA libraries were sequenced on the Illumina GAIIx (76bp PE) (British Columbia Cancer Agency, Vancouver, Canada).

### Transcriptome Assembly

Prior to assembly, all reads were subject to quality control and trimming. The raw reads were trimmed and cleaned with Sickle requiring a minimum read length of 45bp and a minimum quality score of 33 (Joshi 2011). Trimmed reads were subsequently processed with the SolexaQA package to visualize overall quality and remove additional outliers (Cox *et al.* 2010). Following quality control, reads were combined between libraries of the same species and run as a single assembly with default parameters and a minimum contig length of 300bp. The *de novo* transcriptome assemblies were executed with Trinity RNA-Seq (v2.0.2) (Grabherr *et al.* 2011). The Transdecoder tool packaged with Trinity was used to predict coding regions from the transcripts produced in the assembly. The Transdecoder *train* parameter allows the software to learn from previously annotated proteins selected from the same (or closely related) species. Since a comprehensive gene set is presently unavailable for the white pines, 34,059 high quality genes annotated in the loblolly pine genome were used for training (Wegrzyn *et al.* 2014). Additionally, protein domains were considered when homology alone was not sufficient to identify reading frames; version 27.0 of the European Molecular Biology Laboratory’s PFAM-A database was used for this purpose (Bateman *et al.* 2004).

### Structural and Functional Annotation

Custom scripts were used to filter the Transdecoder selected transcripts. First, the longest and highest quality frame was selected from each set of gene products. In addition, full-length genes were curated where a start codon, stop codon, and identifiable protein domain were identified. The most optimal frame for each gene was processed with the EnTAP software package that is designed for non-model transcriptome annotation (http://entap.readthedocs.io/en/latest/introduction.html). The first stage processes local alignments via USEARCH’s ublast package against two NCBI databases (RefSeq and Plant Protein). NCBI BlastX equivalent E-values of 10^−9^ for stringent matches and 0.0001 for weak matches were used. Sources of contamination common to plant tissue, including: insect, fungal, and bacterial contaminants were screened based on stringent matches to annotated proteins. The remaining transcripts and results were converted into an XML file that can be loaded into Blast2GO for Gene Ontology term assignment (Conesa and Götz 2008). Subsequently, the translated transcripts were passed to InterProScan with default parameters to identify conserved protein domains from the Pfam-A database (Jones *et al.* 2014).

### Assembly Validation

To validate the assembled transcripts, GMAP was used to align the sequences to the loblolly pine genome (v2.01) and sugar pine genome (v.1.25) Neale *et al.* 2014; Crepeau *et al.* 2016). GMAP is a splice aware aligner capable of indexing genomes over 20 Gbp in size (Wu and Watanabe 2005). The similarity was assessed in two different runs on each genome: 90% identity/90% coverage and 98% identity/90% coverage.

### Gene Families, Selection, and Candidate Genes

The Markov Cluster Algorithm (MCL) was implemented via TRIBE-MCL to identify shared and unique gene families among the white pine species analyzed (Enright *et al.* 2002). All translated sequences were filtered for a minimum length of 100aa. An all-*vs.*-all BLASTP search was performed using default parameters, followed by clustering with MCL using a moderate inflation value (measure of cluster granularity) set to 4. Data on gene families was filtered and parsed through custom scripts. Functional annotations were merged from EnTAP to further characterize the results.

Multiple sequence alignments of each conserved gene family were prepared with Muscle (Edgar 2010). Alignments were evaluated in Belvu by conservation scores and those with excessive gaps and missing data were filtered (Sonnhammer and Hollich 2005). Pairwise alignments across all four species were evaluated with codeml (PAML, v.4.6) (Yang 2007). The tree file was provided as ((((LP), WWP), WBP), SP) (Parks *et al.* 2009). Synonymous (dS) and nonsynonymous (dN) nucleotide substitution rates per site were calculated using PAML v.4.6. The synonymous/nonsynonymous ratio (dN/dS) is an estimate of natural selection acting on the genes: dN/dS < 1 indicates negative purifying selection, dN/dS = 1 indicates neutral evolution, and dN/dS > 1, indicates positive selection. Alignments with dS values < 0.01 and dS or dN > 2 were discarded. Very high dN/dS (> 10) were also removed as they indicate bias. Candidate gene families involved in drought tolerance and disease resistance were compiled from a number of studies that documented previous genetic associations with these traits of interest in conifers. These were specifically investigated in terms of selection pressure. Functional annotations from EnTAP were queried to identify putative orthologs.

### Data Availability

The following NCBI Bioprojects contain the short reads submitted to Short Read Archive (SRA) as well as the assembled transcripts submitted to the Transcriptome Shotgun Assembly (TSA) database: PRJNA292559, PRJNA254339, PRJNA294917. Table S1 details the gene gain and loss matrix for the four species compared. Table S2 details the annotations available for the gene families unique to each species. Table S3 details the MCL families and their functional annotations. Figure S1 depicts the alignment of positively selected white pine UTP7-like proteins involved in 18S pre-ribosomal RNA processome. Figure S2: depicts an alignment of aridity-associated white pine MATE9-like proteins with three angiosperms (*Vitis vinifera*; *Brassica napus*; *Arabidopsis thaliana*) and Human (*Homo sapiens*) orthologs.

## Results

### Sequencing and Quality Control

Paired-end RNA-Seq was available for all individuals with the exception of sugar pine. Sequencing of western white pine produced 398,534,772 reads which reduced to 208,059,003 after quality control. Whitebark pine produced 1,257,388,110 reads which reduced to 839,389,034 after the quality control pipeline. Sugar pine and limber pine produced 91,223,401 and 669,904,522 reads, respectively. After trimming, sugar pine and limber pine libraries had 66,894,169 reads and 374,191,816 reads, respectively (Table 1).

**Table 1:** Summary of Illumina short read sequencing for the four white pines

**Library**	**Tissue Source**	**Sequencing Technology**	**Reads**	**Total Reads**	**Total Reads (post-QC)**
western white pine	Needle	Illumina GA IIx	PE, 76bp	398,534,772	208,059,003
whitebark pine	Needle	Illumina HiSeq	PE, 100bp	1,257,388,110	839,389,034
sugar pine	Needle	Illumina GA IIx	SE, 80bp	91,223,401	66,894,169
limber pine	Needle	Illumina HiSeq	PE, 100bp	669,904,522	374,191,816

### Transcriptome Assembly and Annotation

The *de novo* transcriptome assembly of sugar pine executed by the Trinity assembler produced a total of 53,821 transcripts, of which 33,533 were identified as unique genes. The mean length and N50 of the contigs were 946 and 1,321, respectively. The limber pine assembly contained 51,694 transcripts, 51,684 of which were unique genes, and had a mean length of 819 with an N50 of 1,067. Whitebark pine and western white pine produced 146,063 and 60,458 transcripts, 145,987 and 49,964 unique genes, had mean lengths of 752 and 800 and N50 values of 1,468 and 1,353 (Table 2). From the unique gene sets, full-length sequences were defined as having annotated start and stop codons, as well as a recognizable protein domain. A total of 16,107 whitebark pine sequences, 4,735 western white pine sequences, 5,000 limber pine sequences, and 3,927 sugar pine sequences were annotated as full-length (Table 3).

**Table 2:** Summary of Trinity *de novo* transcriptome assembly statistics

**Species**	**Total Number of Transcripts**	**Total Number of Genes**	**N50 (All)**	**Median Length (bp)**	**Mean length (bp)**	**Assembly (bp)**	**N50 of Longest Isoform**	**Median of Longest Isoform (bp)**	**Mean of Longest Isoform (bp)**
WWP	60,458	49,964	1353	465	800.46	48,394,102	1271	412	741.58
WBP	146,063	145,987	1468	369	751.68	109,792,255	1468	369	751.57
SP	53,821	33,533	1321	651	945.83	50,905,778	1312	615	922.39
LP	51,694	51,684	1067	559	819.16	42,345,743	1067	559	819.09

**Table 3:** Summary of assembled transcripts annotated as partial and full-length unigenes

**Library**	**Total Number of Sequences (Transdecoder)**	**N25**	**N50**	**N75**	**GC (%)**	**Total Number of Sequences (Non-Contaminated)**	**Total Number of Sequences (Full Length Transcoder)**	**N25**	**N50**	**N75**	**GC (%)**
WBP	23,932	2915	1722	1227	43.88	23,862	16,107	2769	1821	1323	43.96
WWP	10,534	2055	1473	1107	44.13	10,494	4,735	2040	1500	1161	44.63
LP	14,288	1941	1362	1020	44	14,238	5,000	2157	1530	1161	44.45
SP	10,395	2088	1464	1086	44.04	9,362	3,927	2223	1572	1188	44.4

**Figure 2:**
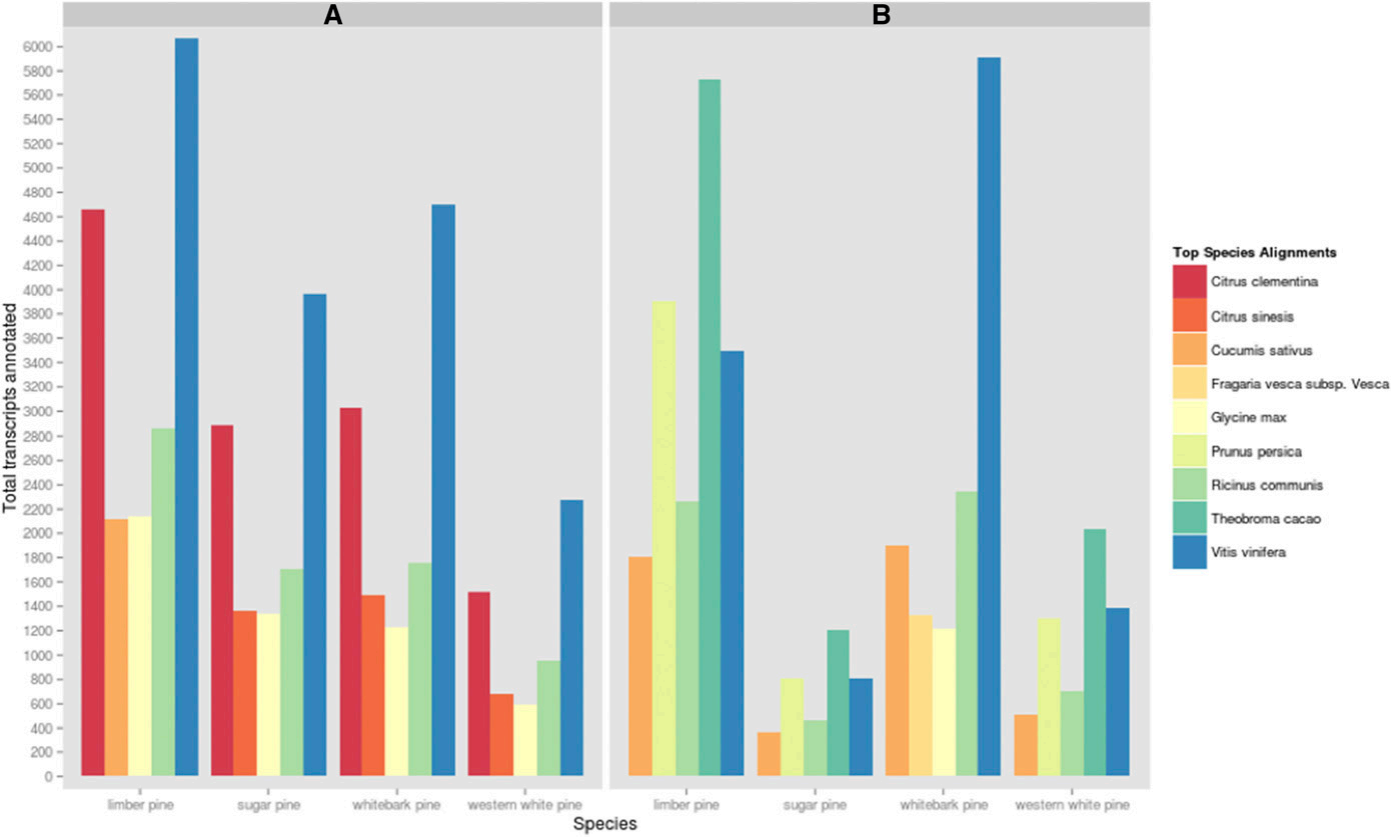
Functional Annotation by Species Homology. A: Top five species sharing annotations with white pine transcriptome assemblies based on the NCBI curated plant full-length protein database as derived from Usearch results. B: Top five species sharing annotations with white pine transcriptome assemblies based on the NCBI RefSeq database as derived from Usearch results.

**Figure 3:**
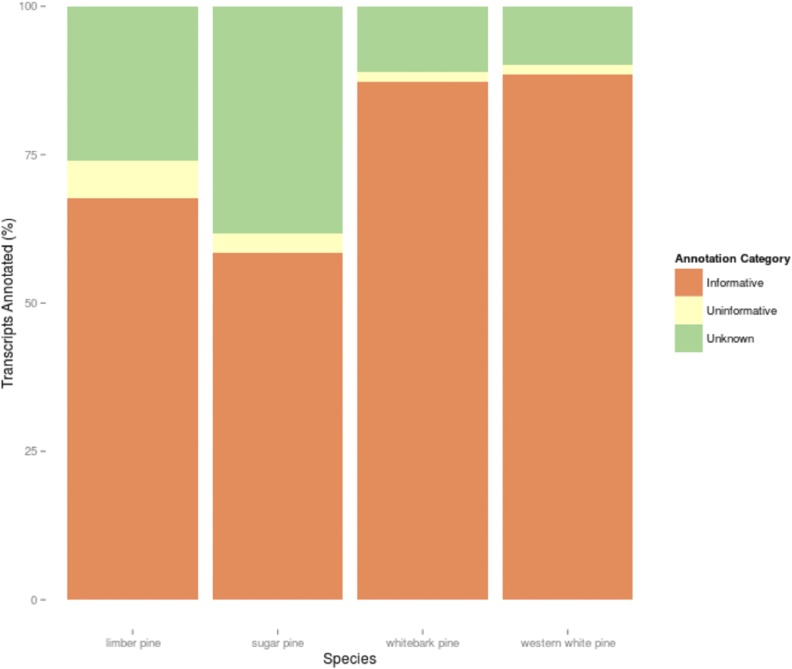
Annotation Quality Summary. Normalized quantity of assembled transcripts that were classified by the EnTAP annotation pipeline for each species as: uninformative (significant alignment that does not contain descriptive function), informative (significant alignment that does contain a descriptive function), or unknown (no significant alignment observed).

The results of the combined annotation through EnTAP which included BLAST searches, Gene Ontology term assignment and InterProScan domain comparisons yielded annotation rates between 60% (sugar pine) and 75% (western white pine). Sequences from Unigene sets were classified as unknown, uninformative, or informative (Figure 3). *Informative* sequences were defined as those sequences which had a significant and descriptive protein annotation. *Uninformative* sequences were sequences confirmed as proteins, but annotated in public databases with unknown functions. *Unknown* sequences were returned without a match. The annotation pipeline selected the most informative match available within the coverage and E-value thresholds specified. The annotation pipeline processed 51,656 queries originating from Trinity-identified genes for limber pine, 23,932 for western white pine, 37,395 for sugar pine, and 145,438 hits for whitebark pine. The annotated transcripts were most commonly shared with nine other fully sequenced angiosperm species. When annotations were compared against the plant protein database, *Vitis vinifera*, *Citrus clementina*, and *Ricinus communis* were the three top matching species (Figure 2A). Interestingly, all white pines included in this study shared annotations with *Citrus sinesis*, with the sole exception of limber pine, in which *C. sinesis* was outmatched by *Cucumis sativus*; more than 2100 limber pine annotations are shared between limber pine and *C. sativus*. Comparison of functional annotation against the NCBI RefSeq database, yielded far less uniform results across species (Figure 2B). Here, *Theobroma cacao* composed the plurality of matches among the four white pines, except whitebark pine, in which *V. vinifera* claimed the bulk of these alignments. In comparison to other white pines, limber pine showed a relatively high number of annotations shared with *Prunus persica*. Shared annotations with *Glycine max* were unique to whitebark pine.

As a result of comparing transcript annotations against fungal, bacterial, and insect filters, some transcripts were identified as contaminants. These are likely associated with the needle tissue during RNA extraction and library preparation. In all species assembled, the primary contaminants included *Neofusicoccum parvum* and *Aureobasidium pullulans*, both plant-associated fungi. Homology comparison using the NCBI RefSeq database identified 771 (0.53%) whitebark transcripts, 30 (0.26%) western white transcripts, 46 (0.12%) sugar pine transcripts, and 86 (0.17%) limber pine transcripts as contaminants which were removed after annotation.

### Assembly Validation

**Figure 4:**
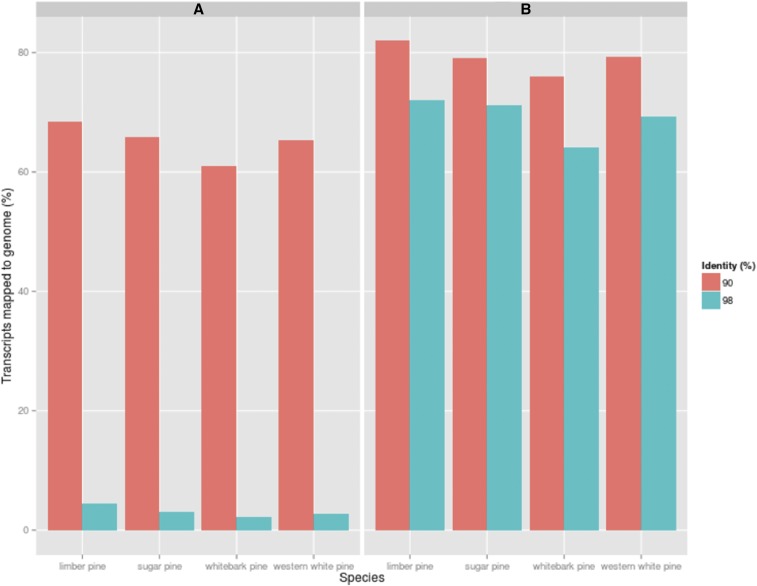
Transcripts Aligned to Conifer Reference Genomes. A: Percent of assembled transcripts by species mapping back to loblolly pine reference genome at 90% identity/90% coverage and 98% identity/90% coverage. B: Percent of assembled transcripts by species mapping back to sugar pine reference genome at 90% identity/90% coverage and 98% identity/90%coverage.

The *de novo* constructed assemblies were used to evaluate spliced alignments of genes against the loblolly pine and recently published sugar pine genome to assess homology and validate the transcriptome assembly. The results of a GMAP run requiring 98% identity/90% coverage returned low alignment percentages, averaging 3.12%. In total, this represents 2.77% of western white pine genes, 2.15% of whitebark genes, 3.10% of sugar pine genes, and 4.47% of limber pine genes. With a much lower threshold of 90% identity/90%coverage, the alignments increase dramatically; 65.23% of western white pine genes, 61.08% of whitebark pine genes, 65.80% of sugar pine genes, and 68.34% of limber pine genes (Figure 4A). This analysis was repeated with the sugar pine genome assembly. The lower threshold run requiring 90% identity/90%coverage resulted in 79.32% of western white pine, 76.07% of whitebark pine, 79.17% of sugar pine, and 81.98% of limber pine genes aligning. The most stringent parameters which required the same coverage and 98% identity yielded alignments averaging 70% for all four species (Figure 4B). Assemblies aligned with the sugar pine genome at a more stringent threshold (98%), while alignment to loblolly pine was successful at a lower threshold (90%). This difference can be accounted for by evolutionary distance. Sugar pine, like the white pines analyzed here, is of the subgenus *Strobus*, while loblolly pine is of the subgenus *Pinus*. *Pinus pinus* and *Pinus strobus* are roughly 45 million years diverged, accounting for the variation in alignment strength (Wang and Wang 2014).

### Gene Families, Selection, and Candidate Genes

**Figure 5:**
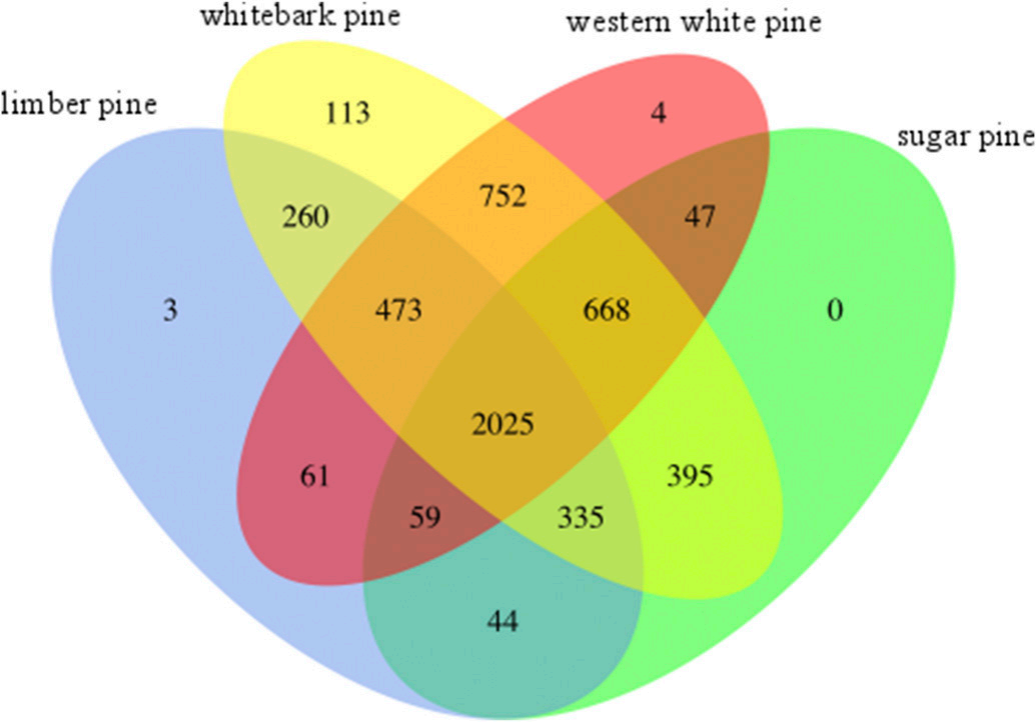
Orthologous Gene Families. TRIBE-MCL evaluation of a total of 58,148 translated transcripts reveals shared and unique gene families. Integer counts in the Venn indicate number of unique families shared between each combination for white pine proteins. A total of 2,025 gene families were conserved across all four species.

The orthologous protein analysis was implemented via TRIBE-MCL on a total of 58,148 protein sequences: 14,288 from limber pine, 9,394 from sugar pine, 23,862 from whitebark pine, and 10,534 from western white pine. These sequences formed 6,782 (11.7%) unique gene families, of which 5,239 (77.2%) had at minimum two proteins per family and were included in subsequent analysis. The 1543 singlets, families to which exactly one sequence was assigned, were removed from the analysis. A total of 3,488 (western white pine: 768, sugar pine: 616, whitebark pine: 1593, limber pine: 511) identified families had at least two total members, which could have been distributed across any permutation of species. Protein domain annotations were assigned to the proteins prior to clustering into families (Table S3). The predominant PFAM domains were variations of PF00069 and PF13041, which represent the protein kinase domain (15 families representing 356 genes) and the pentatricopeptide repeat (PPR) family (4 families representing 475 genes), respectively. The analysis also identified gene families exclusive to each species. These include three for limber pine, 113 gene families for whitebark pine, and four for western white pine. None were identified as unique to sugar pine. The unique families ranged in size from 2 to 5 members (Table S2). A total of 2,025 gene families was shared among all white pines in this study. These families ranged in size from 4 to 505 sequences. Western white pine and whitebark pine represent the two species that shared the most gene families; 752 families were shared exclusively between those two species (Figure 5; Table S1).

**Figure 6:**
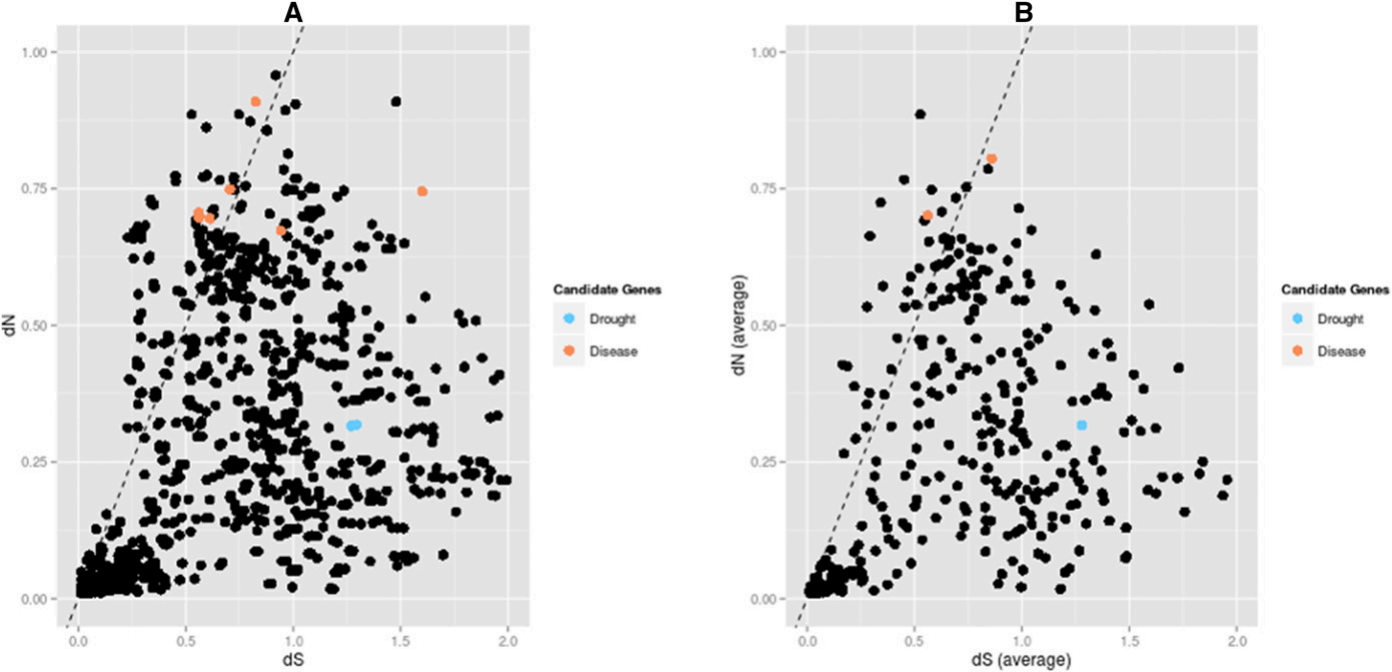
Distribution of dN and dS. A: Pairwise alignments across the four species for 408 gene families. dN/dS values > 1 indicating positive selection are shown above the dashed line. Candidate genes with previous associations to drought tolerance/aridity and rust resistance are highlighted. B: Averaged values for dN/dS across all species for each of the 408 gene families. dN/dS values > 1 indicating positive selection are shown above the dashed line. Candidate genes with previous associations to drought tolerance/aridity and rust resistance are highlighted.

From the conserved set of 2,025 gene families across all four species, Muscle was used to align 2,022 viable sequence sets. Filtering of the alignments reduced this number to 1,467. From here, the dN and dS values were filtered, removing those that were less than 0.01 or greater than 2. dN/dS values that were greater than 10 were also removed. This resulted in pairwise alignments for a total of 408 genes (Figure 6A). Of these 408, 39 are estimated to be under positive selection (average dN/dS > 1), 9 are under neutral selection (average dN/dS < 1 and average dN/dS > 0.95), and the remaining 360 are under purifying selection (average dN/dS <0.95) (Figure 6B, Table 4).

**Table 4:** Summary of conserved gene families under positive selection

**Gene Family Annotation**	**Alignment Length**	**Gene Ontology (Molecular Function)**
formyltetrahydrofolate deformylase mitochondrial isoform x1	1053	formyltetrahydrofolate deformylase activity; amino acid binding; hydroxymethyl-, formyl- and related transferase activity;
f-box kelch-repeat protein skip6-like	1122	protein degradation tagging activity
low quality protein: nitrate reductase	2778	oxidoreductase activity; metal ion binding; organic cyclic compound binding; heterocyclic compound binding
arogenate dehydrogenase chloroplastic	1278	prephenate dehydrogenase activity
carrier protein chloroplastic	2283	ATP:ADP antiporter activity; ATP binding
flowering time control protein fpa	3279	
transcription initiation factor tfiid subunit partial	1818	
e3 ubiquitin-protein ligase keg isoform x2	4917	protein degradation tagging activity
PREDICTED: uncharacterized protein LOC18435046	1524	
two-component response regulator-like prr37	2856	
isoamylase chloroplastic	2769	
probable u3 small nucleolar rna-associated protein 7	1623	18S ribosomal rna processing
PREDICTED: uncharacterized protein LOC103493568	1407	metal ion binding;sequence-specific DNA binding transcription factor activity
calcium-transporting atpase plasma membrane-type-like isoform x1	3192	calcium-transporting ATPase activity; calmodulin binding; ATP binding; metal ion binding
protein notum homolog	1263	
PREDICTED: uncharacterized protein LOC104607701	1380	
clathrin assembly protein at5g35200	1644	1-phosphatidylinositol binding; clathrin binding
PREDICTED: kanadaptin	2274	
family 18 glycoside hydrolase	1236	chitinase activity; chitin binding
dead-box atp-dependent rna helicase 13	1176	
probable inactive purple acid phosphatase 27	1977	acid phosphatase activity; metal ion binding; dephosphorylation
arginine decarboxylase	2283	carboxy-lyase activity
PREDICTED: uncharacterized protein LOC104591536	1626	
erythronate-4-phosphate dehydrogenase-like protein	975	
interferon-induced guanylate-binding protein 2-like	3207	GTPase activity; GTP binding
nf-x1-type zinc finger protein nfxl1	4290	metal ion binding
transmembrane protein 87b-like	1560	
fructokinase-like chloroplastic	1644	kinase activity; phosphotransferase activity, alcohol group as acceptor
bel1-like homeodomain protein 1	2517	DNA binding
cbs domain-containing protein cbsx6	1311	
duf21 domain-containing protein at4g14240	1623	
probable wrky transcription factor 14	1431	
myeloid leukemia factor 1-like isoform x2	1059	
mannose-1-phosphate guanylyltransferase 1	948	
phytoene synthase chloroplastic	1308	geranylgeranyl-diphosphate geranylgeranyltransferase activity; phytoene synthase activity
unknown	336	
PREDICTED: myosin-10-like	1803	
universal stress protein a-like protein	366	
PREDICTED: uncharacterized protein LOC104602728	966	

**Figure 7:**
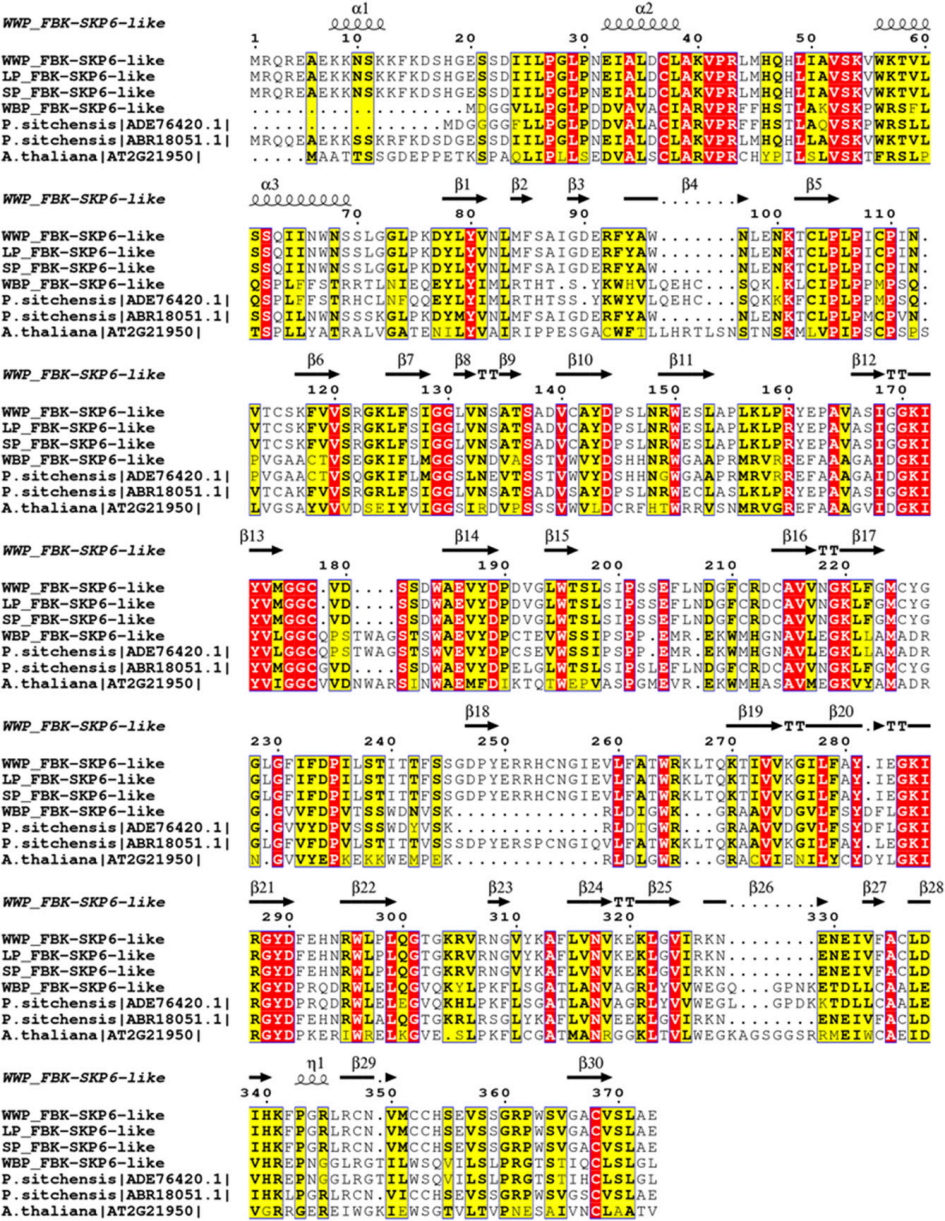
Alignment of FBK-SKiP6-like proteins estimated to be under positive selection and associated with WPBR response in conifers. White pine FBK-SKiP6-like proteins are aligned against sequences available in sitka spruce (*Picea sitchensis*) and Arabidopsis. In the alignment, red regions are 100% conserved residues, yellow is conserved at 70% identity or greater, and black regions represent similar residues. Secondary structure is based on WWP protein model generated by I-Tasser. The alignment was generated by ESpript (Robert and Gouet 2014).

Although not positively selected, several gene families with documented associations with drought tolerance and rust resistance in conifers were identified in the conserved gene families. One drought associated gene was characterized from two Mediterranean pines, *P. pinaster* and *P. halepensis: 4-coumarate CoA ligase* (*4CoA*) (Grivet *et al.* 2011) and three aridity associated genes from loblolly pine: RING/U-box superfamily protein, MATE efflux protein 9, UDP-galactose transporter from loblolly pine (Eckert *et al.* 2010) (4CoA:[130], RING/Ubox:[350, 1989, 1032, 690], MATE9: [24], UDP-gal: [1728, 1952]). Two minor genes involved in rust resistance include a receptor-like protein kinase (RLK) homologous to a *Picea glauca* gene and an NBS-LRR gene were characterized in *P. monticola* (Liu *et al.* 2013). Following filters applied for alignment quality and pairwise divergence, the disease resistance gene family (NBS-LRR) characterized originally in *P. monticola* and the drought associated gene (MATE efflux protein 9) characterized originally in *P. taeda* (Figure S2) were included in the gene sets. Pairwise alignments revealed that the NBS-LRR disease protein associated with WPBR resistance was under positive selection (dN/dS > 1) (Figure 6A). Among the positively selected genes, a Kelch motif bearing F-box protein (F-box-SKP6) and E3 ubiquitin ligase (Figure 7, Table 4) are known to be involved in resistance to plant necrotrophic fungi infection through E3-ubiquitin ligase 26S proteasome pathway documented in *Arabidopsis* (Kepinski and Leyser 2005) AVP1: [207], F-box-SKP6: [891], E3ligase: [932], NBS-LRR: [9]) (Figure S3).

## Discussion

Evaluation of the reads and subsequent assemblies highlights differences in the transcriptomes among the four species. Most notably, the limber pine assembly yielded the shortest N50, mean, and median length values, suggesting that the assembler had the greatest difficulty constructing full length contigs from the read data in this species. Whitebark pine, western white pine, and sugar pine produced comparable quality assemblies in terms of the overall length. However, if an assessment were to be made on the assemblies considering the number of genes and full-length transcripts, limber pine fares much better with 14,438 unique genes with 5,000 predicted as full-length. Conversely, the sugar pine assembly generated 33,533 unique genes with only 3,927 annotated as full-length. The whitebark pine assembly yielded the greatest number of full-length genes at 16,107. If evaluated in terms of annotation rates, western white pine and whitebark pine had the lowest percentage of unknowns (1.71% and 1.78%, respectively) and uninformative sequences (9.83% and 11.02%) indicating that the assembled contigs aligned well to annotated proteins. Sugar pine and limber pine annotation did not perform as well. Sugar pine had the highest number of uninformative and unknown sequences (41.8%) and limber pine followed with 32.29%. An increased number of unannotated transcripts can result from artifacts of the assembly process as well as a prevalence of short contigs that cannot be uniquely aligned against public protein databases. Inconsistent experimental designs and sequencing technologies contribute to some of the differences observed in the final *de novo* assemblies (Loman *et al.* 2012). Sugar pine represented three distinct individuals and was sequenced on older Illumina GAIIx technology as single-end reads, which yielded shorter reads. For the other species (whitebark pine, western white pine, and limber pine), paired-end Illumina HiSeq technology was implemented but the needle tissue sampled for RNA sequencing was pooled from multiple individuals. Many conifer species show remarkable diversity, and substantial differences can be observed across relatively small geographic ranges (Hamrick *et al.* 1992). Variation in the total number of genes from transcriptome assemblies may be inflated from pooled samples as individual variation is introduced and difficult to reconcile at the assembly stage.

In recent years, several conifer needle transcriptomes have been assembled. One of the first transcriptomes from NGS technology examined the transcriptome from lodgepole pine using the Roche 454 platform and identified 17,000 unique genes (from needle and conelet tissue) with an average contig length of 500 bp (Parchman *et al.* 2010). Differences in sampling strategies, assembly software, and sequencing technologies make comparisons between the assemblies challenging, but the total number of unique genes is similar to the averages observed for the white pines assembled here. Recent needle transcriptomes have ranged from 25,000 to 47,000 unique transcripts with huge variation in assembly software, filtering approaches, and annotation of true genes (Chen *et al.* 2012, Howe *et al.* 2013). Estimates of the total gene space in conifers has recently converged to a range of 30,000 to 45,000 as a result of the availability of genome sequences (Neale *et al.* 2014; Nystedt *et al.* 2013). On average, these transcriptome assemblies yielded fewer unique genes than the whole genome estimates. This is expected as these assemblies represent only needle tissue.

Variation in *de novo* transcriptome assembly is heavily influenced by the assembly software selected. The open source software packages for constructing transcriptomes includes: Trans-ABySS, Velvet/Oasis, SOAPdenovo, and Trinity. It is unlikely that each of these would create assemblies of equal caliber. The Liu *et al.* (2013) assembly for western white pine used the commercial CLC Workbench for the first published assembly. For sake of consistency and to optimize the comparative analysis, the western white pine transcriptome was reconstructed here with Trinity. While there is presently no available literature comparing the efficacy of these assemblers in gymnosperms, some work has been done to compare assemblers in angiosperms and other species which led to the selection of this tool. Zhang *et al.* (2013) evaluated transcriptome assembler performance on geraniums and found clear evidence that Illumina sequencing data paired with the Trinity assembler produced the best results in the absence of a reference genome. Trinity has also been shown to outperform other *de novo* assembling technologies in terms of short term read mapping and assembly of complete contigs (Clarke *et al.* 2013). Similarly, Zhao *et al.* (2011) found that Trinity assemblies were of superior quality to other *de novo* assemblers in a study that compared performance on *Camellia sinensis*, *Drosophila melanogaster*, and *Schizosaccharomyces pombe*.

Orthologous gene family analysis estimates shared and unique families across the transcriptomes assembled. Where possible, MCL gene families were paired with protein domain information as annotated through the Pfam database. As expected, the 2025 gene families shared among all four pine species were largely associated with the most highly conserved functions. The largest gene family, consisting of 505 members, relates to antifungal properties which is of great interest in relation to rust resistance. The majority of gene members contain a combination of protein kinase and stress domains which together have a role in salt stress response and fungal resistance. The protein kinase domain associated with over 500 families is one of the most conserved domains across eukaryotes (Manning *et al.* 2002). It is implicated in various essential autonomic processes including apoptosis, metabolism, signal transduction, among others (Manning *et al.* 2002). The PPR repeat family, represented in 4 families with a total of 464 sequences is often amplified in plant species via retrotransposition and is common in plant organellar proteins (O’Toole *et al.* 2008). It has been shown that the proliferation of this family in plants has a deep evolutionary link to the highly complex RNA metabolism functions of organelles (Barkan and Small 2014). Also, highly represented in the conserved families, are the Deaminase and DNA binding domains. Frigida, a family of proteins associated with regulation of flowering time and vernalization response, is present equally in the four white pines studied; three sugar pine and western white families and four limber pine and whitebark pine families are annotated (Preston and Sandve 2013). Flowering regulation is an important vernalization activity, so the Frigida family identified here likely indicates the strong vernalization response. This may be especially true given their alpine habitat and need to reproduce after the conclusion of a harsh winter. Strong vernalization responses often indicate strong cold tolerance, as well as cold acclimation (Preston and Sandve 2013). These features make Frigida an interesting candidate for abiotic stress.

The species presented in this study with the highest number of potentially unique families was whitebark pine, to which 113 families were predicted. Functional annotation of 13 families indicates that the 100 potentially unique families identified with no assigned protein domain information may be completely novel protein families in whitebark pine. Among the 13 annotated families, two (*Reverse Transcriptase 1*[823] and *Reverse transcriptase 3*[1197]) are likely to be retrotransposons. The remaining families have functions associated with drought tolerance and other forms of abiotic stress response including disease resistance (MAP) and UV resistance (*impB C-terminal domain*[5194]). The *microtubule associated family*[199] (MAP) has been shown to play a role in resistance to tobacco mosaic virus (Ashby *et al.* 2006, Xiong and Yang 2003). Although the role of MAP in conifers is unclear, these genes may be involved in disease resistance. Limber pine and western white pine produced a similar number of unique gene families with three and four, respectively. Just one of the four families identified exclusively in western white pine was associated with protein domain information. The *gag-polypeptide of LTR copia-type*[5118] domain, which, like the reverse transcriptase domains, is associated with retrotransposons. In limber pine, one family annotated as *Exocyst complex component Sec6*[4290] was present. This domain is well characterized in angiosperms for its role in vesicle docking (Cole *et al.* 2005). Needle tissue has high levels of photosynthetic activity and it seems likely that the Sec6 domain plays a role in vesicle docking associated with photosynthesis and related processes.

From the highly conserved genes identified, a set of 408 were aligned and examined for signatures of selection. Among these, 39 were under positive selection and the majority of these (33) were functionally described as transcription factors, chloroplast proteins, and various protein kinases (Table 4). Much of our understanding of fungal disease resistance comes from biotrophic pathogens where auxin signaling sits at the heart of plant-pathogen interactions (Fu and Wang 2011). As a biographic pathogen that causes indirect damage by causing a sink in the infected tissue fatally altering allocation of photosynthetic assimilates, major genes conferring resistance to WPBR originates from different sources including chitinases, calcineurin B-like (CBL)-interacting protein kinases (CIPK), abscisic acid (ABA) receptor; transcriptional factor (TF) genes of multiple families; genes homologous to apoptosis-inducing factor (AIF), flowering locus T-like protein (FT), subtilisin-like protease and F-box family proteins (FBP) (Liu *et al.* 2011, Liu *et al.* 2013). Among these, an F-box protein, was detected as positively selected (Table 4). F-box proteins are subunits of E3 ubiquitin ligase aggregations called SCF quaternary complex (SKP1, Cullin1, F-box protein and Rbx1) (Zheng *et al.* 2002). Cullin1 forms the backbone of the complex holding the F-Box-SKP1 (S-phase kinase associated protein) in its N-terminal. F-Box-SKP1 forms a dimer with a multitude of other F-Box-SKP proteins with different C-terminal protein-protein interaction domains including WD40 repeat, the Leucine-rich repeat, Tub, Lectin and Kelch repeats increasing the combinatorial diversity. The *Arabidopsis* genome encodes 10 Cullins, 21 *Skp1*-like genes and more than 700 F-Box proteins (Risseeuw *et al.* 2003) which can tag a large repertoire of proteins for degradation through 26S Ubiquitin-proteasome protein degradation pathway. In western white pine, 22 related differentially expressed genes after WPBR infection were involved in the 26S Ubiquitin-proteasome pathway and 14 of them were up-regulated only in resistant seedlings (Liu *et al.* 2010). In Arabidopsis, auxin binding to the SCFs leads to degradation of transcriptional repressors belonging to the AUX/IAA family through the SCF E3-ubiquitin ligase proteasome (26S) pathway. The degradation of the AUX/IAA transcriptional repressors leads to the expression of auxin-responsive genes, which, trigger plant resistance to necrotrophic fungi (Kepinski and Leyser 2005). Strikingly, E3-ubiquitin-protein ligase is also among the positively selected gene set (Table 4).

One of the protein interaction domains in F-box proteins is the Kelch repeat domain which is annotated in one family across all four species and under positive selection (Table 4; Figure 7). The whitebark pine ortholog of this protein was different from the rest of the white pines and showed significant homology with one of the two sitka spruce orthologs (Figure 7). The paralog of FBK-SKiP6 showed homology to western white pine, limber pine and sugar pine (Figure 7). The F-box proteins harboring Kelch motif (KFB) contains 44–56 amino acid residues and was first identified in *Drosophila* (Xue and Cooley 1993; Bork and Doolittle 1994). Kelch repeat sequence motifs correspond to 4-stranded antiparallel beta-sheets. In whitebark pine and western white pine, F-Box-SKP6 proteins harbor 5 Kelch motifs forming a super-barrel structural arrangement known as a beta propeller (Figure S3). KFBs have expanded in plant lineages through multiple duplication events with varying number of Kelch motifs ranging from one to five. In *Arabidopsis*, KFBs have been found in tandemly duplicated copies (up to 95 copies) and in loblolly pine 10 homologs are known to exist (Sun *et al.* 2007). *Arabidopsis* KFBs (ZTL, FKF, LKP2) are involved in the flowering time and circadian control (Nelson *et al.* 2000; Han *et al.* 2004; Somers *et al.* 2004; Yasuhara *et al.* 2004; Imaizumi *et al.* 2005). Plant KFBs may have expanded and diverged functionally from that of animals and may have gained additional functions on top of the protein degradation pathway.

Essential for 18S ribosomal subunit biogenesis, the UTP7-like protein is under positive selection when examined across the four white pines (Table 4, Figure S1). The hierarchy of ribosome biosynthesis is quite high among other metabolic processes since it is the key element of the Central Dogma. The processome of the 18S pre-ribosomal RNA requires 28 proteins to allow U3 snoRNA to excise and fold the subunit. U3-associated reactions take place early in ribosome biogenesis. The 35S pre-ribosomal RNA transcript is the largest ribonucleoprotein complex known in the living world and the first three cleavages separate 18S, 5.8S and 26S rRNA molecules. These proteins do not have catalytic functions but their binding is a prerequisite for base pairing interactions of the U3 snoRNA with the 5′ ETS 18S pre-ribosomal RNA. Depletion of UTP7 in a yeast experimental system was shown to prevent U3 snoRNA scaffolding activity (Dragon *et al.* 2002). Intriguingly, UTP7 has been shown to be present in kinetochores where chromosomes are segregated during cell division (Jwa *et al.* 2008). Western white pine appears to express an angiosperm-like ortholog of UTP7 from differing from those of other white pines and sitka spruce (Figure S1). Western white pine seedlings probably express juvenile version of the UTP7 homologous to those of angiosperms adapted to meet the demands of fast dividing cells. The observed difference from the remaining white pines may be a result of the age of needle tissue used for cDNA library preparation.

Drought response is a complex trait in plants and involves contributions from multiple genes. In our set of conserved genes across all species, we identified four genes (4CoA, MATE9, RING/U-box, UDP-gal transporter) that were previously associated with aridity and drought. We, however, did not detect any signature of selection among them.

## Conclusion

This study represents a comparative analysis of *de novo* transcriptome assemblies for four non-model white pine species. Deep Illumina sequencing was used to assemble normalized needle RNA tissue libraries in order to characterize both rare and abundant transcripts. Assembled genes were highly conserved when evaluated as orthologous groups with over 2000 of the 5239 gene families shared among all four species. In examining sequence alignments of these conserved genes, we identified 39 genes under positive selection, some of which are associated with traits of interest such as disease resistance and drought tolerance. Prior to this study, very few genetic resources existed for this group of five-needle pines whose populations have been severely threatened by WPBR, mountain pine beetle and the related effects of a changing climate. The transcriptomes described here provide a foundation for understanding the underlying molecular interactions in these species. Genes that are under positive selection and also implicated in disease resistance or drought tolerance serve as potential targets for breeding programs, which can further select for these favorable traits. This will serve to improve the efficiency of breeding programs aimed at restoring threatened populations, and protect the ecological and economic value of the white pines.

## Supplementary Material

Supplemental Material is available online at www.g3journal.org/lookup/suppl/doi:10.1534/g3.118.200257/-/DC1.
