# Supplementary figures and images for: Comparative Transcriptomics Among Four White Pine Species
Source: G3 (Bethesda). 2018 Mar 27;8(5):1461–74. doi: 10.1534/g3.118.200257 (PMC5940140; doi:10.1534/g3.118.200257)

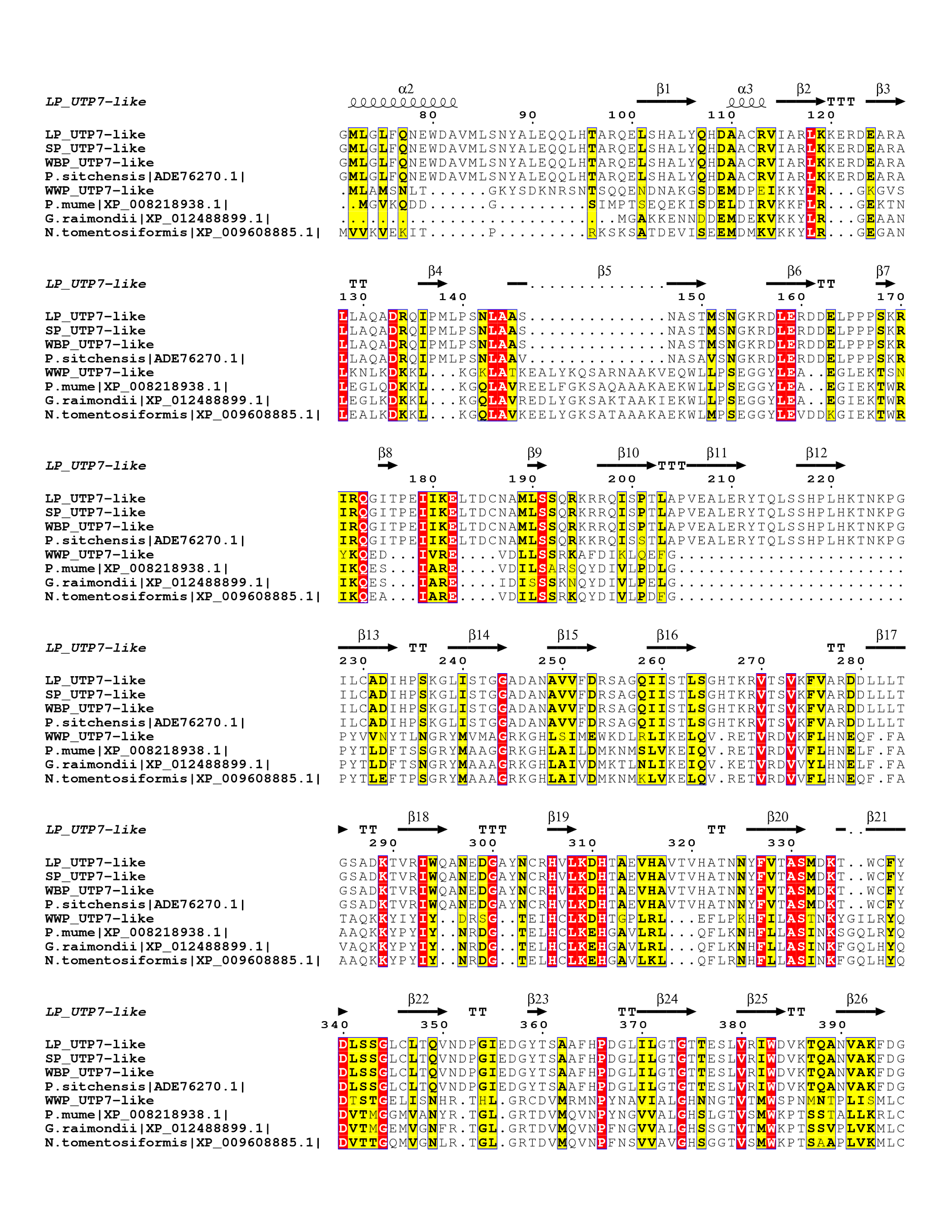
Figure S1 (family 938 UTP7-like in two parts)


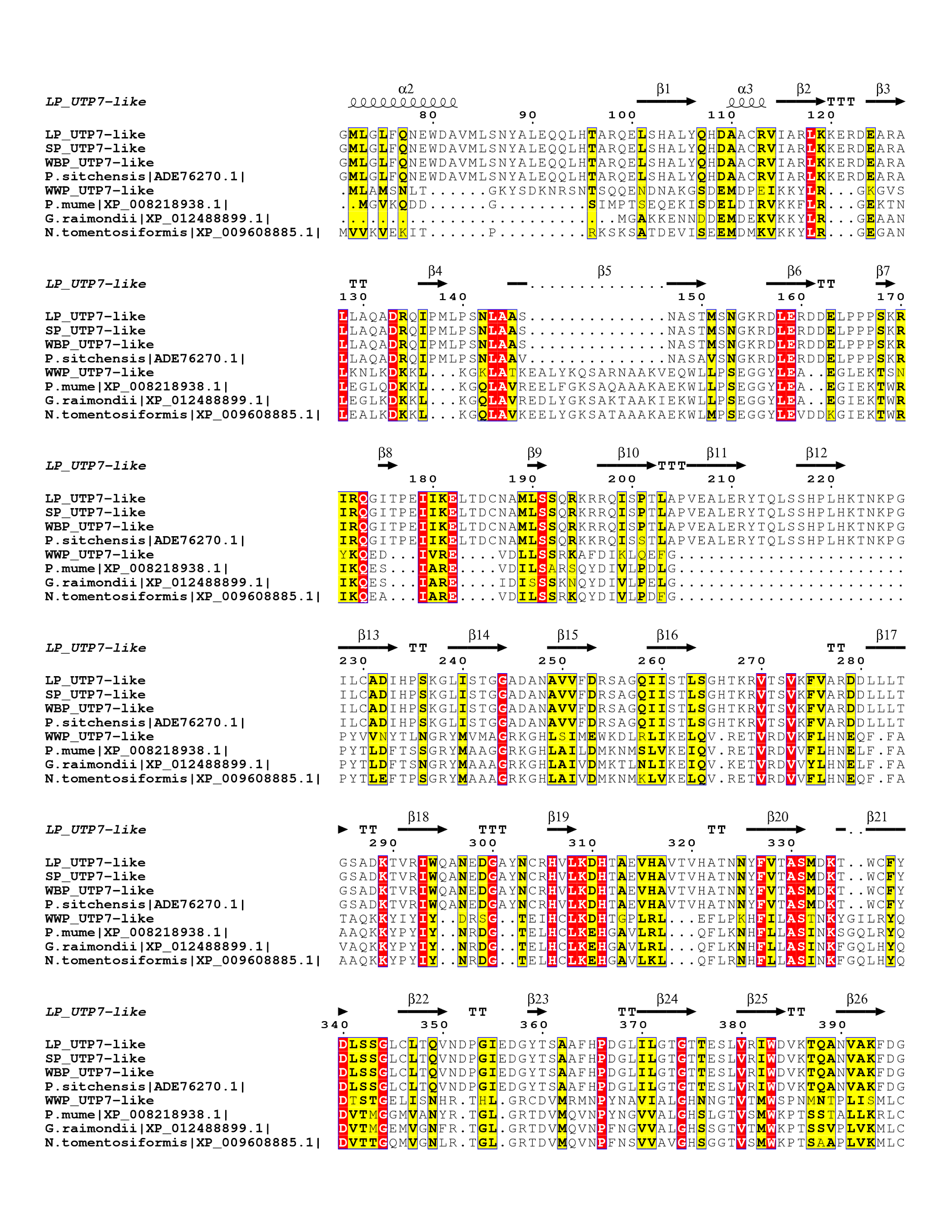


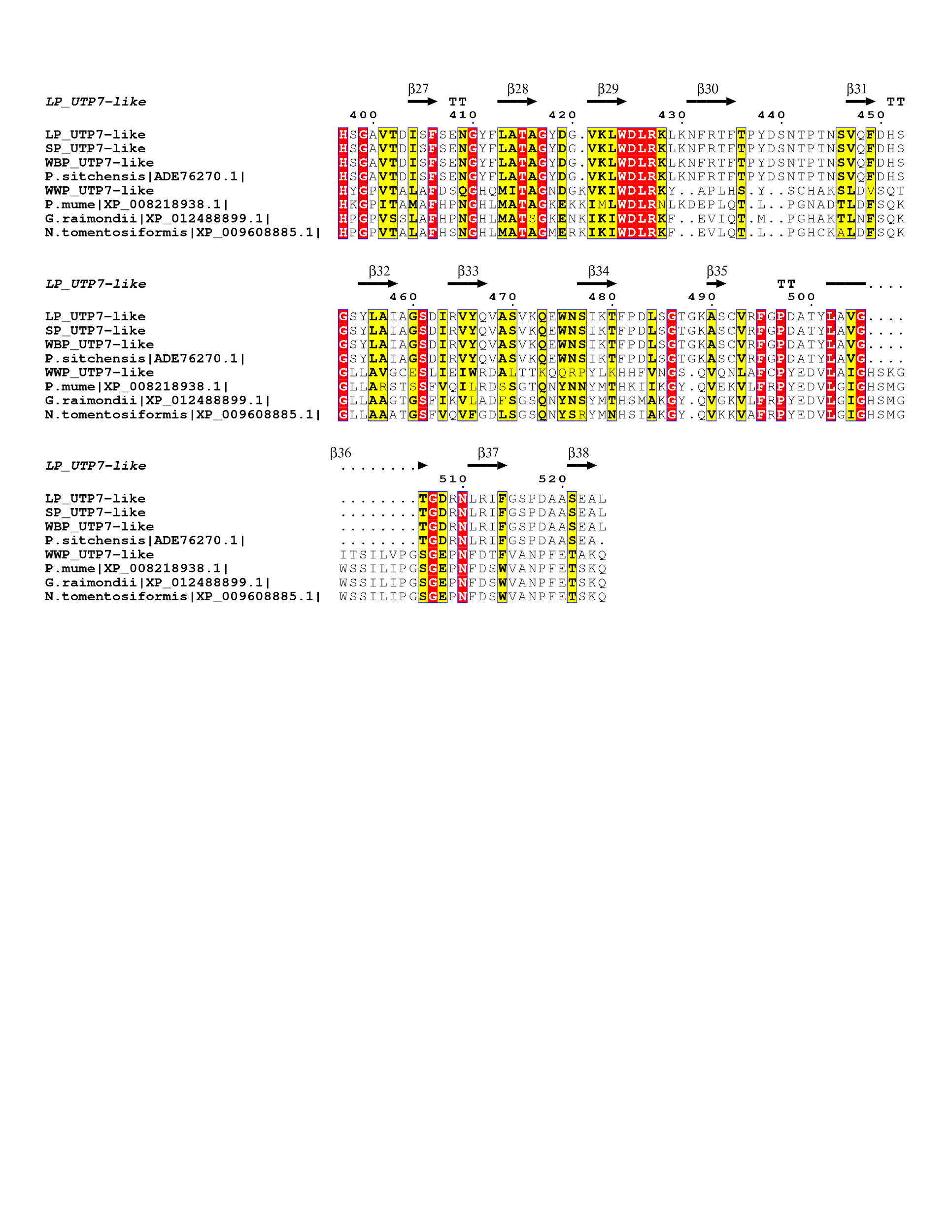

Supplement: Supplementary file 1 [file 1461FigureS1.docx]

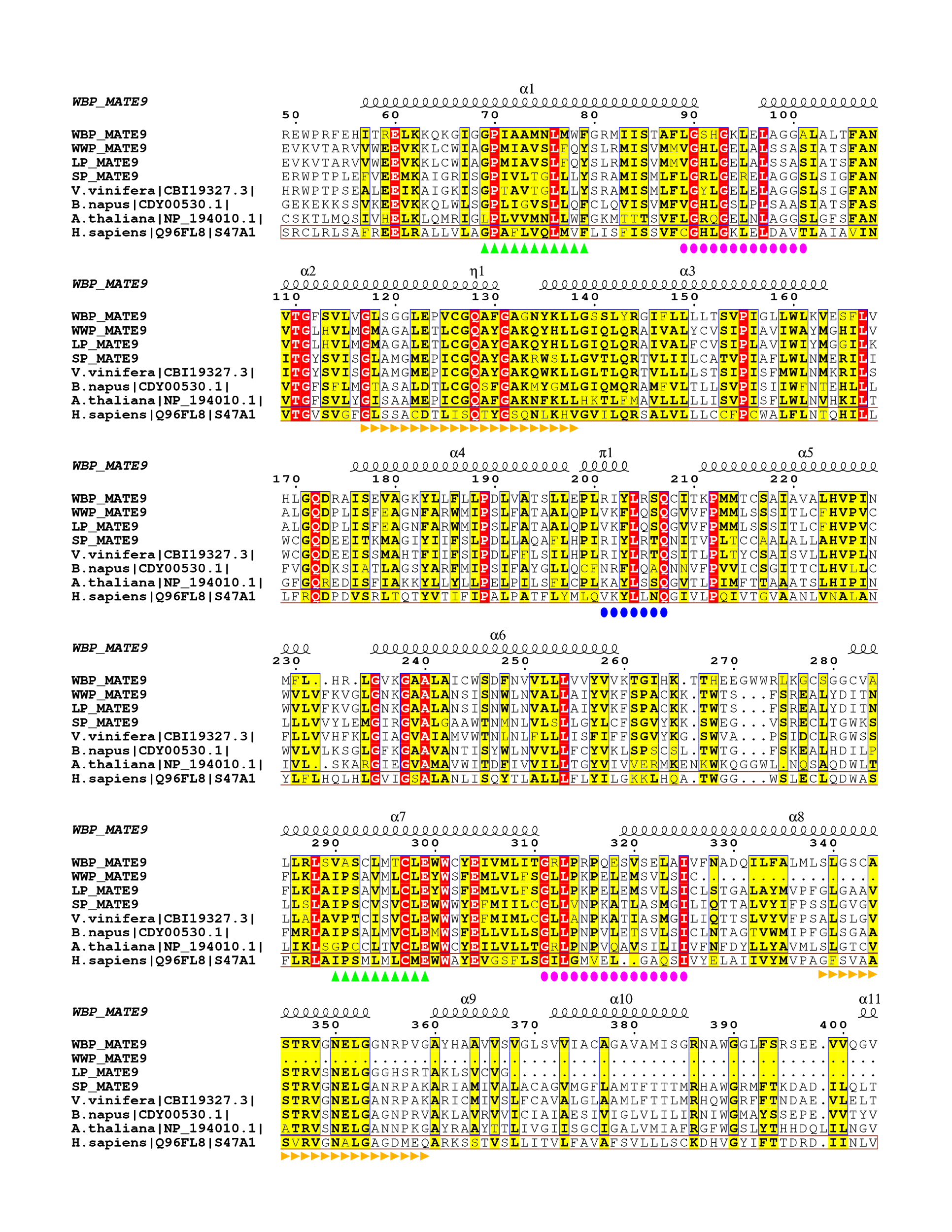

Supplement: Supplementary file 2 [file 1461FigureS2.docx]
