# Supplementary material for: Comparative Transcriptomics Among Four White Pine Species
Source: G3 (Bethesda). 2018 Mar 27;8(5):1461–74. doi: 10.1534/g3.118.200257 (PMC5940140; doi:10.1534/g3.118.200257)
Supplement: Supplementary file 3 [file 1461FigureS3.docx]

Figure S3: **Video of the positively selected WBP (left) and WWP (right) FBK-SKiP6 proteins showing their conserved and non-conserved domains colored the same way as in Figure 7.** A section of the conserved domains (residues 181-190 in WWP, 168-177 in WBP) shown in ball and stick representation for visual reference. Proteins are rotated to expose propeller-like Kelch repeat domains that play role in binding to the Ubiquitin protein degradation pathway which ultimately leads to fungal pathogen resistance. <https://vimeo.com/117768143> (password: bioinformatics)
