# Supplementary material for: Comparative Transcriptomics Among Four White Pine Species
Source: G3 (Bethesda). 2018 Mar 27;8(5):1461–74. doi: 10.1534/g3.118.200257 (PMC5940140; doi:10.1534/g3.118.200257)
Supplement: Supplementary file 4 [file 1461TableS1.docx]

Table S1: Summary of gain and loss of specific gene families across the four sequenced white pines

|  | **limber pine** | **western white pine** | **sugar pine** | **whitebark pine** |
| --- | --- | --- | --- | --- |
| **limber pine** | 0 | -829 | -313 | -1761 |
| **western white pine** | 829 | 0 | 516 | -932 |
| **sugar pine** | 313 | -516 | 0 | -1448 |
| **whitebark pine** | 1761 | 932 | 1448 | 0 |
