# Supplementary material for: Comparative Transcriptomics Among Four White Pine Species
Source: G3 (Bethesda). 2018 Mar 27;8(5):1461–74. doi: 10.1534/g3.118.200257 (PMC5940140; doi:10.1534/g3.118.200257)
Supplement: Supplementary file 5 [file 1461TableS2.docx]

Table S2: Summary of unique gene families organized by species with protein domain annotations

| **Species** | **Genes in Family** | **PfamId[PfamCount]:PfamDescripton** | **Description** |
| --- | --- | --- | --- |
| **limber Pine** | 2 | PF06046[1]:Sec6 | Exocyst complex component Sec6 |
| **whitebark Pine** | 3 | PF00013[1]:KH_1 | KH domain |
|  | 2 | PF00078[1]:RVT_1 | Reverse transcriptase |
|  | 2 | PF00153[1]:Mito_carr | Mitochondrial carrier protein |
|  | 2 | PF00488[1]:MutS_V | MutS domain V, DNA mismatch repair |
|  | 2 | PF01007[1]:IRK | Inward rectifier potassium channel |
|  | 2 | PF01066[1]:CDP-OH_P_transf | CDP-alcohol phosphatidyltransferase |
|  | 3 | PF04193[1]:PQ-loop | PQ loop repeat |
|  | 2 | PF11799[1]:IMS_C | impB/mucB/samB family C-terminal domain |
|  | 2 | PF12214[1]:TPX2_importin | Cell cycle regulated microtubule associated protein |
|  | 3 | PF12706[1]:Lactamase_B_2 | Beta-lactamase superfamily domain |
|  | 5 | PF13456[1]:RVT_3 | Reverse transcriptase-like |
|  | 2 | PF13532[1]:2OG-FeII_Oxy_2 | 2OG-Fe(II) oxygenase superfamily |
|  | 2 | PF13639[1]:zf-RING_2 | Ring finger domain |
| **western white pine** | 2 | PF14223[1]:UBN2 | gag-polypeptide of LTR copia-type |
